# Core-Shell Structured Carbon@Al_2_O_3_ Membrane with Enhanced Acid Resistance for Acid Solution Treatment

**DOI:** 10.3390/membranes12121246

**Published:** 2022-12-09

**Authors:** Qianlian Wu, Huimiao Zhang, Yi Zhou, Zhishu Tang, Bo Li, Tingming Fu, Yue Zhang, Huaxu Zhu

**Affiliations:** 1 Jiangsu Collaborative Innovation Center of Chinese Medicinal Resources Industrialization, Nanjing University of Chinese Medicine, Nanjing 210023, China; 2 Jiangsu Botanical Medicine Refinement Engineering Research Center, Nanjing University of Chinese Medicine, Nanjing 210023, China; 3 State Key Laboratory of Research & Development of Characteristic Qin Medicine Resources, Shaanxi University of Chinese Medicine, Xianyang 712038, China; 4 China Academy of Chinese Medical Sciences, Beijing 100700, China

**Keywords:** acid resistant, alumina membrane, carbon coating, adsorption, kinetic and thermodynamic

## Abstract

Ceramic membrane has an important application prospect in industrial acid solution treatment. Enhancement of the acid resistance is the key strategy to optimize the membrane treatment effect. This work reports a core–shell structured membrane fabricated on alumina ceramic substrates via a one-step in situ hydrothermal method. The acid resistance of the modified membrane was significantly improved due to the protection provided by a chemically stable carbon layer. After modification, the masses lost by the membrane in the hydrochloric acid solution and the acetic acid solution were sharply reduced by 90.91% and 76.92%, respectively. Kinetic models and isotherm models of adsorption were employed to describe acid adsorption occurring during the membrane process and indicated that the modified membrane exhibited pseudo-second-order kinetics and Langmuir model adsorption. Compared to the pristine membrane, the faster adsorption speed and the lower adsorption capacity were exhibited by the modified membrane, which further had a good performance with treating various kinds of acid solutions. Moreover, the modified membrane could be recycled without obvious flux decay. This modification method provides a facile and efficient strategy for the fabrication of acid-resistant membranes for use in extreme conditions.

## 1. Introduction

Ceramic membranes are increasingly gaining attention due to their high mechanical strengths, thermal stabilities, high resistance to contamination, and long service lives [1,2,3,4,5]. As a result, they are widely used in oil–water separations, wastewater treatment, pharmaceutical production, and food processing [6,7,8,9,10]. Ceramic membranes generally have sandwich structures consisting of a top separation layer, a transition layer, and a support layer [11]. The mechanical strength of the ceramic membrane is dominated by the support [12,13,14], which is fabricated by calcining raw material power with additives [15,16,17]. The glass phase produced during calcination is an important factor affecting the mechanical strength of the ceramic membrane, since it acts as the binder [18,19]. Therefore, the stability of the support depends on the raw material and the glass phase. In addition, the support layer has an influence on the membrane performance [20] due to its nonnegligible thicknesses. Because the internal transport channels were formed by the stacking of the ceramic particles, which were bonded by additives [12,20,21]. So, the characteristics of the internal transmission channel affect the mass transfer process. Alumina has commonly been used as the support layer for various functionalized ceramic membranes, such as polymer-coated composite ceramic membranes, zeolite molecular sieve membranes, and graphene oxide membranes, due to its wide availability, low cost, proven preparation processes, and so on [22,23,24,25]. Now, increasing numbers of researchers are devoting their efforts to studies of the support layer [26,27].

Acidic solutions are common in production and processing chains, such as pharmaceutical production, food processing, textile dyeing, and metal smelting. Membrane technology has been applied to a large number of inorganic and organic acids [28,29,30,31]. Compared to neutral systems, acidic solutions require a higher stability for the separation membranes. However, given that defects usually accumulate at oxide grain boundaries, hydrogen ions enter the vacant sites of the lattice and combine with oxygen negative ions, which often causes the dissociation of metal ions [32,33]. As an amphoteric metal oxide, alumina may be susceptible to acid corrosion, which mainly occurs with inter-crystalline phases and additive-produced glass phases [32,34]. For example, Ma et al. found that the corrosion resistance was improved as the proportion of Al_2_O_3_/SiO_2_ increased, especially in an acidic environment [34]. This might result in the deterioration of the membrane strength and dealumination and introduces impurities into the permeate [35,36]. On the other hand, the acidic components may be adsorbed by coordination with the abundant hydroxyl groups on alumina [21] during the production and processing of natural herbal extracts and juices rich in acidic active ingredients, which may result in irreversible loss of acidic active ingredients and destruction of the flavors [28,29]. To improve the stabilities of alumina membranes and reduce adsorption, various methods have been used to modify alumina ceramic membranes, such as surface coating with raw micro/nanoparticles and doping with suitable additives, to modulate the active sites [37,38,39]. However, there are struct requirements in term of the size and uniformity of the insoluble micro/nanoparticles, as well as the stability of the suspensions with the micro/nanoparticles dispersed in the solvent. Moreover, various additional chemical reagents are required for modification, which makes recycling difficult. Thus, drawbacks such as complex preparation processes, difficult control of conditions, and irreversible damage have limited the application and expansion of these methods [35].

Biochar is widely used because of its simple preparation, high chemical stability, abundant active functional groups, and limited adsorption of acidic components, which can be removed by a simple annealing process [40,41]. The carbon coating is produced on various substrates by the hydrothermal carbonization of biochar, regardless of their shape and surface morphology [42]. Carbon coating has been applied on porous ceramics in many areas. Due to its excellent mechanical properties such as hardness, fracture toughness, and bonding strength, the carbon coating is usually used as a wear-resistant layer [43,44]. It is also commonly used to deal with residual stress in ceramic and metal joints to protect the mechanical properties of joints between them [45,46]. The good corrosion resistance and oxidation resistance of the carbon coating has enabled it to be applied in rocket equipment [47,48]. The permeability and selectivity of the ceramic membrane is improved with the carbon coating, which can be prepared by the one-step spray carbonization method [49] and the carbonization of PFA followed by pencil coating [50]. A large number of active functional groups on the surface of the carbon layer can regulate the interaction between the solid substrate and the solute [51]. Moreover, it has been reported that the carbon coating functions as a protective layer. For example, Zhang et al. [52] reported that the carbon layer coated on the surface of SiC protected the boron carbide from dissolution. Moreover, Xu et al. [53] reported that the corrosion resistance of a chemically bonded phosphate ceramic with modified MWCNTs was improved. This provided us with the inspiration to develop a non-destructive and effective method for improving the stabilities of ceramic membranes used to treat acidic solutions.

To fabricate the acid-resistant membrane, glucose was used as the carbon source for a simple hydrothermal reaction designed to form a carbon-coated alumina ceramic membrane (C@ACM). The C@ACM showed an obvious core–shell structure, and the carbon shell effectively enhanced the resistance to acid. Compared to the ACM, the induced oxygen-containing functional groups on C@ACM adjusted the electrical interactions and hydrogen bonding between the acid and the membrane, resulting in lower acid depletion. Additionally, thermodynamic and kinetic adsorption models were applied to describe the acid depletion process. The cycling test further confirmed that C@ACM exhibited better adsorption resistance than ACM alone. The flux of the recycled membrane was consistent with that of the ACM, which indicated that C@ACM is highly recyclable. This work may pave the way for the facile preparation of acid-resistant membranes with applications in extremely acidic environments.

## 2. Materials and Methods

### 2.1. Chemicals and Materials

Al_2_O_3_ membranes (flat sheet, pore size ≈ 150 nm, thickness ≈ 2 mm, effective area = 0.000314 m^2^) were purchased from Nanjing Gaoqian Functional Materials Technology Co., Ltd., Nanjing, China. Glucose (AR) was purchased from Nanjing Shoude Reagent Co., Ltd., Nanjing, China. Hydrochloric acid (HCl, 36.0~38.0%) was purchased from Shanghai Lingfeng Chemical Reagent Co., Ltd., Shanghai, China. Acetic acid (HAc, 99.5%) was purchased from Nanjing Chemical Reagent Co., Ltd., Nanjing, China. Oxalic acid (99.0%), DL-tartaric acid (99.0%), L-malic acid (99.0%), citric acid (99.5%), gallic acid (99.0%), and salvianolic acid B (80.0%) were purchased from Nantong Feiyu Biotechnology Co., Ltd., Nantong, China. Chlorogenic acid (98.0%) was purchased from Ark Pharm (Arlington Heights, IL, USA). Sulfuric acid (95~98%) was purchased from Xilong Chemical Reagent Co., Ltd., Shantou, China. The lab-made deionized water was used throughout the whole experimental work.

### 2.2. Fabrication of C@ACM

The C@ACM was fabricated on alumina ceramic substrates by a one-step in situ hydrothermal method. Briefly, the ACM was placed vertically in the Teflon container, with the homogenous glucose aqueous (0.5 mol·L^−1^, 30 mL) followed by a 30 s ultrasonic treatment and a 12-h soakage to be well immersed. Then, they were transferred to the matching reaction kettle and heated at 180 °C for 8 h in an oven. After cooling to room temperature (25 °C), the membrane was rinsed repeatedly with ultrapure water and ethanol until the leaching solution was colorless, and then dried at 50 °C.

### 2.3. Characterization

**Figure 1:**
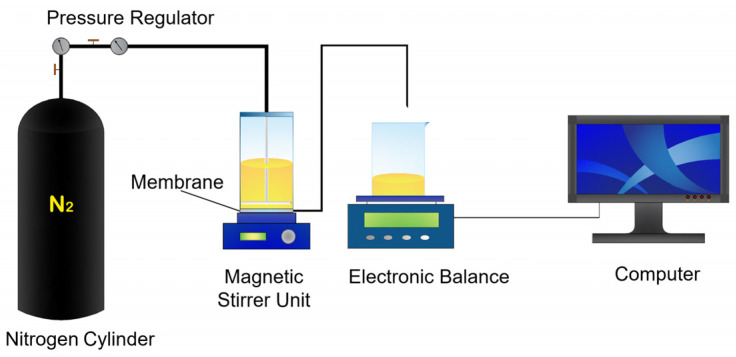
The schematic diagram of the ceramic membrane cross-flow filtration dead-end filtration.

The pure water permeance was tested using deionized water by a dead-end filtration apparatus equipped with external pressure supplied by high-purity nitrogen gas (Figure 1). The permeance (*J*) can be calculated by the following Equation (1):

(1) $$J=\Delta V∕S_{ef}tP\times 100\%$$

where ΔV is the total volume of the permeation, $S_{ef}$ is the effective area, t is the operation time, and P is the trans-membrane pressure.

The membrane morphology and element mapping images were imaged by SEM (QUANTA FEG 250, Hillsboro, OR, USA) with EDX. The internal structures of ACM and C@ACM were imaged by TEM (HT7800, Hitachi, Tokyo, Japan). Mercury intrusion porosimetry (MIP) (AutoPore IV 9500, Tekronix, Beaverton, OR, USA) was used to detect the membrane pore size and distribution. The surface composition and chemical states of ACM and C@ACM were detected by XPS (AXIS, Shimadzu, Kyoto, Japan) using Al Kα radiation. Raman spectrums were performed with LabRam HR Evolution (HORIBA, Paris, France) using an excitation wavelength of 532 nm. The surface chemistry was measured by ART-FTIR (Nicolet iS5, Thermo Fisher Scientific, Waltham, MA, USA). The crystalline phases of ACM and C@ACM materials were identified using an X-ray diffraction (XRD) instrument (Empyrean, Panalytical, Almelo, The Netherlands) equipped with a Cu-Kα radiation source (λ = 0.154 nm) operated at 45 kV and 40 mA. The scans were performed over a 2*θ* range from 5° to 90°. Water contact angle measurement (FCA2000A, AFES) was employed to characterize ACM and C@ACM.

### 2.4. Acid Resistance Test

The acid resistance was evaluated by the weight loss of the dry membranes after the acid solution treatment. The ACM and C@ACM were immersed in the hydrochloric acid (HCl) and acetic acid (HAc) solutions with pH = 3.50 at 80 °C for 24 h, respectively. After that, the membranes were taken out of the solutions and washed with deionized water until the pH of the washing water was neutral. A lower mass loss means better acid resistance.

### 2.5. Adsorption during the Filtration Process and Model Fitting

Considering that alumina is a kind of metal oxide, the instability of the alumina ceramic membrane may be related to acid corrosion with the consumption of hydrogen ions. To further explore the discrepancy in the mechanism of acid consumption on ACM and C@ACM, the adsorption kinetics and dynamics models were applied to describe the whole process, which was evidence of the enhanced acid resistance of C@ACM. Hydrochloric acid, the simplest acid, was taken as an example for the experiment. Batch filtration experiments were conducted to investigate the effects of concentration and time, with the dead-end filtration apparatus (Figure 1). The membrane was placed at the bottom of the cup. The operating pressure during the filtration was 0.2 MPa. The temperature was controlled at 25 °C. The electronic balance and the computer were employed to record the weight of the permeate solution. The feed and the permeates were collected. The concentration of the feed and the permeates were determined by sodium hydroxide titration. Conductivity and pH were two indicators to measure the change of the solution before and after the membrane process, which were measured by a conductivity analyzer (DDS-307A, Leici, Shanghai, China) and a pH meter (PB-10, Sartorius, Gottingen, Germany), respectively. The penetration was calculated by Equation (2) based on the titration results:

(2) $$P=c_{P}∕c_{f}\times 100\%$$

where P (%) is the penetration and $c_{P}$ and $c_{f}$ (mmol·L^−1^) are the concentration of the permeation and the feed, respectively. The consumption of acid calculated by the titration results was further applied to the adsorption kinetic and isotherm studies.

#### 2.5.1. Adsorption Kinetics

The kinetic data were analyzed by pseudo-first order, pseudo-second order, the Weber and Morris model, and the Elovich model. The pseudo-first-order and the pseudo-second-order kinetic model are given as Equations (3) and (4), respectively:

(3) $$\ln\left(q_{e}-q_{t}\right)=-K_{1}t+\ln q_{e}$$

(4) $$t∕q_{t}=1∕K_{2}q_{e}^{2}+t∕q_{e}$$

where $q_{t}$ and $q_{e}$ (mmol·cm^−2^) are the amount of adsorbed acid per square centimeter at time t and the equilibrium time, respectively. $K_{1}$ (min^−1^) and $K_{2}$ (cm^2^·mmol^−1^·min^−1^) are the pseudo-first and second-order model rate constants, respectively. The Weber and Morris model (W–M model), also called the internal diffusion model, is another classical adsorption kinetic model. Generally, the adsorption process includes three steps: film diffusion, intraparticle diffusion, and pore diffusion. The W–M model works when the resistance in film diffusion can be neglected and the diffusion direction is random. Here, the adsorbate concentration was considered not to have changed with the particle position. The W–M model is given as Equation (5):

(5) qt=kipt12+C

where $k_{ip}$ is the rate constant of the internal diffusion process. C is a constant related to the thickness and the boundary layer.

The Elovich model is usually used to describe the adsorption behavior of pollution on the surface of heterogeneous solids. The Elovich model can be written as Equation (6) and transferred to (7):

(6) $$q_{t}=\ln\left(1+\alpha \times \beta \times t\right)∕\beta$$

(7) $$q_{t}=\left(\ln t+\ln\alpha \beta \right)∕\beta$$

where α is the initial rate constant and β is a constant related to the surface coverage of the adsorbent and activation energy of chemisorption. Moreover, $q_{t}$ (mmol·cm^−2^) and t (min) in Equations (5)–(7) are the same as those in Equations (2) and (3).

#### 2.5.2. Adsorption Isotherm

The adsorption capacities to ACM and C@ACM were evaluated by fitting the experimental data to the following isotherm models at 298 K. The Freundlich model describes multilayer adsorption on a heterogeneous surface of the adsorbent where the energy level of the adsorption site is not constant. This empirical model is represented by Equation (8):

(8) $$lg\;q_{e}=\mathrm{lg}c_{e}/n+lg\;k_{F}$$

where $c_{e}$ (mmol·L^−1^) is the equilibrium concentration of the acid, $q_{e}$ (mmol·cm^−2^) is the amount of the adsorbed acid at the equilibrium point; $k_{F}$ (L·cm^−2^) and *n* are the Freundlich constants, expressing the adsorption capacity and the intensity of the adsorption, respectively. Furthermore, the value of 1/n in two ranges of 0.1 < 1/n < 0.5 and 1/n > 2 implies favorable and unfavorable adsorption, respectively.

The Langmuir model is based on the monolayer adsorption on the surface of a homogeneous adsorbent with constant energy levels. The equation is represented as Equations (9) and (10):

(9) $$c_{e}∕q_{e}=ce∕q_{max}+1∕k_{L}q_{\max}$$

(10) $$\theta =q∕q_{max}=q_{max}∕\left(1+k_{1}c\right)$$

where $q_{max}$ is the maximum of the adsorbent and $k_{L}$ is the Langmuir adsorption equilibrium constant. The value of $k_{L}$ is positively related to adsorption capacity. θ is the coverage of adsorbed molecules on the surface of the adsorbent, q is the amount of the adsorbent, $k_{1}$ is the ratio of the adsorption rate constant to the desorption rate constant, and c is the concentration of acid. When the concentration of acid is very small, $k_{1}$ can be ignored. Then the equation is expressed in Equation (11):

(11) $$q=q_{m}k_{1}c=k^{\prime }c$$

Equation (11) resembles the Henry model, which is expressed as Equation (12):

(12) q=Hc

where c (mmol·L^−1^) is the concentration of the acid, q (mmol·cm^−2^) is the amount of the adsorbent acid, and H (L·cm^−2^) is the constant to express the adsorption capacity under a fixed temperature. In the Henry model, the amount of adsorbent is directly proportional to the concentration of acid.

Equation (13) resembles the Tempkin model:

(13) $$q_{e}=RT\ln\left(ac_{e}\right)∕b$$

which can be represented as Equation (14):

(14) $$q_{e}=B\ln c_{e}+A$$

The Radke–Prausnitz model is represented as Equation (15):

(15) $$q_{e}=abc_{e}^{\beta }/\left(a+bc_{e}^{\beta -1}\right)$$

In Equations (13)–(15), $q_{e}$ (mmol·cm^−2^) and $c_{e}$ (mmol·L^−1^) are equilibrium adsorption capacity and equilibrium concentration. In Equations (13) and (14), T (K) is the temperature and R, a, b, A, and B are both Tempkin constants. In Equation (15), a, b, and β are constants for the Radke–Prausnitz model.

### 2.6. Membrane Performance

#### 2.6.1. Penetration of Acid Components

To explore the acid penetration performance of the membranes, eight types of acids, including acetic acid, oxalic acid, malic acid, tartaric acid, citric acid, gallic acid, chlorogenic acid, and salvianolic acid B were employed for filtration tests with the dead-end filtration apparatus (0.2 MPa, 25 °C) mentioned above. The concentration was 0.5 mmol·L^−1^ for each. The feed and permeation were collected.

A high-performance liquid chromatography-tandem UV detector (Waters e2695-2998, Milford, MA, USA) equipped with a ZORBAX SB-C18 column (4.6 mm × 250 mm, 5 μm, Agilent, Santa Clara, CA, USA), was employed for content determination of the gallic acid, chlorogenic acid, and salvianolic acid B. The mobile phases were acetonitrile (solvent A) and 0.2% phosphoric acid in ultrapure water (*v*/*v*, solvent B). The isocratic elution ratios were 7% A with 93% B for gallic acid, 13% A with 87% B for chlorogenic acid, and 30% A with 70% B for salvianolic acid B, respectively. The UV detection wavelengths for gallic acid, chlorogenic acid, and salvianolic acid B were 270 nm, 327 nm, and 286 nm, respectively. The column temperature was 28 °C and the flow rate was 1 mL·min^−1^. A total of 10 μL of each sample was injected into the analyzing system automatically. The rest of the acid concentrations were determined by sodium hydroxide titration. The acid penetrations were calculated by Equation (1). At the same time, the pH and conductivity of the feeds and permeates were measured.

#### 2.6.2. Performance of the Acid Extract Solution

An extract solution rich in acid components was applied for the membrane performance test. Briefly, about 40 g of hawthorn was boiled twice with 400 mL water for half an hour each time, and then the final volume was adjusted to 400 mL. The feed was obtained after centrifugation (8000 rpm, 10 min), and then it was applied for the membrane filtration tests (0.2 MPa, 25 °C). The membrane filtration experiments were ended until the volume of the permeate reached 60 mL. The membrane performance was evaluated by the permeance and penetration of titratable acid as well as the pH and turbidity of the feed and the permeates.

### 2.7. Coating Stability Evaluation and Recyclability Verification

The stability of the carbon coating layer was evaluated by the pH value change after the five-cyclic filtration of the hydrochloric acid solution with pH = 3.5. After each cycle of filtration, the membrane was rinsed thoroughly with DI water for 24 h until the permeate was neutral. The low pH value change reflected the good stability of the carbon layer. Finally, the recyclability of C@ACM was verified by the water flux recovery after calcination in the muffle furnace at 550 °C for 4 h.

## 3. Results

### 3.1. Characterization of ACM and C@ACM

Pure water permeance was tested under 0.2 MPa, and the change in the permeance is depicted in Figure S2. The results show that the pure water permeance of ACM and C@ACM was maintained at 487.87 and 375.67 L·m^−2^·h^−1^·MPa^−1^, respectively. The decreased permeance of C@ACM can be attributed to the carbon layer, which narrowed the pore size and reduced the flow rate of the liquids.

**Figure 2:**
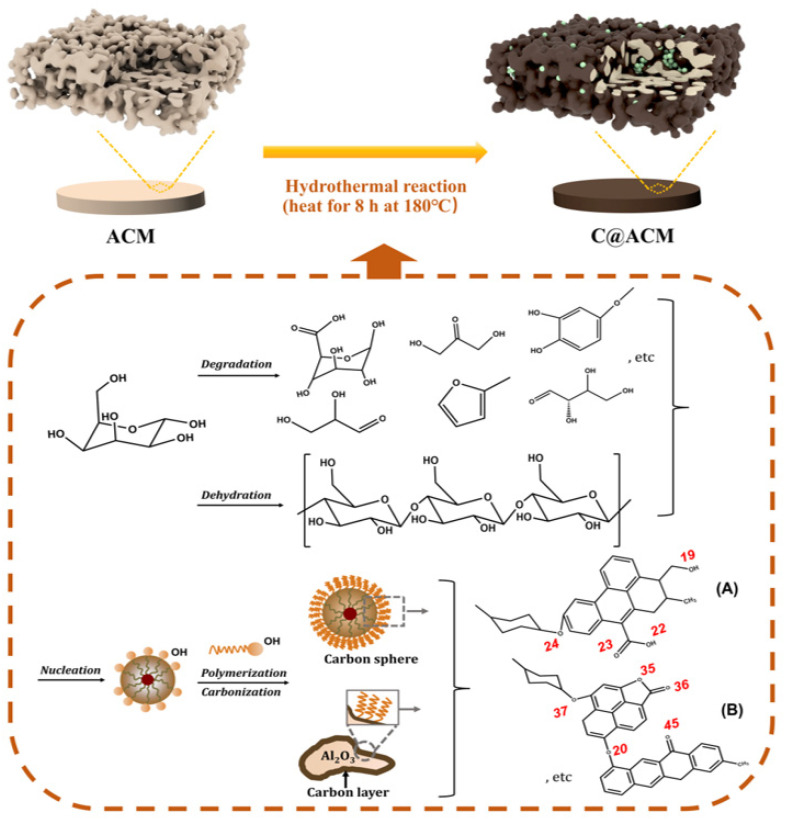
Fabrication of C@ACM by the hydrothermal method and the reaction mechanism. (The white porous structure represents the ACM, the black shell represents the carbon layer, and the green spheres represent the carbon spheres. A and B were the typical products, and they were further applied in the Milluiken charges calculation with Gaussian 09W software).

To obtain the acid resistance membrane, the chemically stable carbon was chosen as a protection layer. As shown in Figure 2, glucose degraded and dehydrated at high temperatures, and then gradually polymerized and carbonized to form a carbon layer covering the surface, and a small amount of the polymerized carbon spheres scattered in the pores of the ACM.

**Figure 3:**
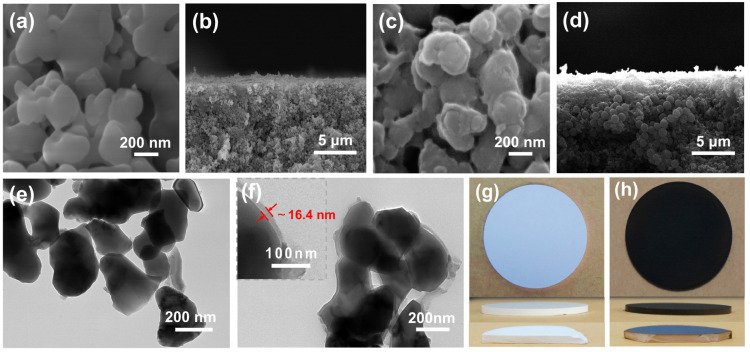
Images of ceramic membranes: (**a**,**b**) SEM images of ACM and (**c**,**d**) C@ACM ((**a**,**c**): surface; (**b**,**d**): cross-section); (**e**) TEM images: alumina particles; (**f**) carbon-coated alumina particles; (**g**,**h**) Photography images of ACM and C@ACM (1-top; 2-side; 3-cross-section).

**Figure 4:**
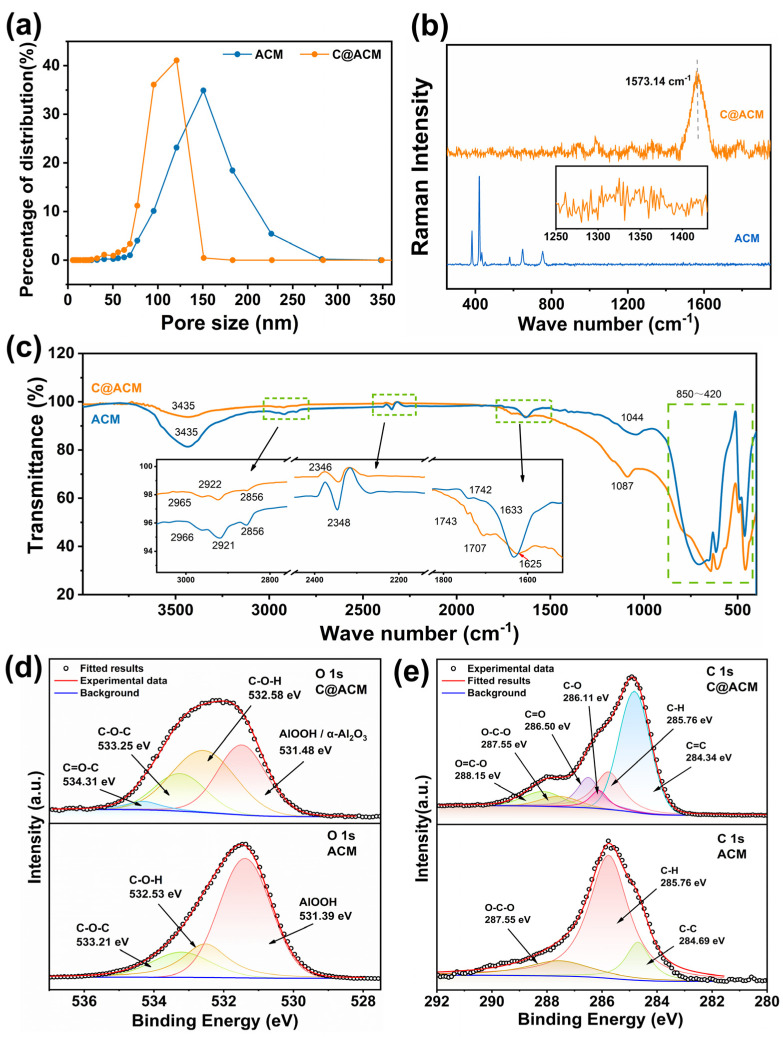
Characterization of ACM and C@ACM: (**a**) pore size distribution; (**b**) Raman spectra; (**c**) FT-IR spectra; (**d**,**e**) XPS spectra of O 1s and C 1s.

The morphology of ACM and C@ACM was confirmed by SEM. The ACM exhibited a typical symmetric porous structure (Figure S1), and the surface of the ACM was relatively smooth (Figure 3a,b). After being coated with carbon, the morphology of C@ACM (Figure 3c,d) changed. Firstly, the carbon layer coated on the alumina particles formed a core–shell structure with a rougher surface. The TEM image (Figure 3e,f) further confirmed the core–shell structure and indicated that the thickness of the carbon layer was about 16.4 nm. For this reason, the membrane pore size might have reduced. The membrane performance might have changed due to the active groups induced by the carbon layer. Secondly, the carbon spheres (Figure 3d) accumulated in the large pores and filled the defects, resulting in the reduction in large pore size and a more uniform pore size distribution. The pore size distribution curves well confirmed this (Figure 4a). The average pore size decreased from 148.93 nm (ACM) to 107.48 nm (C@ACM) after modification. Moreover, the pore sizes larger than 150 nm were significantly reduced, and the pore size distribution was narrowed.

After the modification, the color of the membrane changed from white to brown (Figure 3g,h), and the color of the outer surface was darker than the cross-section. This revealed that the carbon content of the outer surface may have been higher than the cross-section. To further verify this, both the inner and outer surfaces of C@ACM were coated with carbon and the composition of ACM and C@ACM on the surface and cross-section elements were tested by EDS. As shown in Table 1, after coating, the carbon contents of the surface and cross-section of C@ACM were 16.25% and 9.62%, respectively, which was significantly higher than those of the ACM surface (2.78%) and cross-section (0.82%). Meanwhile, the aluminum contents decreased from 43.23% (surface) and 44.54% (cross-section) of ACM to 36.60% (surface) and 34.56% (cross-section) of C@ACM, respectively. This revealed that a carbon layer was successfully coated on the alumina surface. The higher carbon content of the outer surface could be attributed to the horizontal placement of the ceramic membrane, which can provide more stable support for glucose carbonization. Moreover, the EDS results of the hydrothermal precipitate showed that the precipitate consisted of 24.72% O atom and 75.28% C atom, indicating the O-containing groups with a relatively high C/O ratio may have existed on the modified membrane surface.

As has been reported [54,55], the D and G peaks in Raman spectra are the characteristic peaks of carbon materials, which are associated with disordered and ordered graphitic structures, respectively. The Raman spectra of C@ACM (Figure 4b) showed an broad and strong G peak around 1573.14 cm^−1^ and a weak D peak around 1325 cm^−1^, indicating that a typical amorphous carbon structure feature was obtained. The peaks from 400 to 800 cm^−1^ disappeared because the alumina substrate was covered by the carbon layer. Information about functional groups on the membrane surface was given by FT-IR spectra (Figure 4c). The peaks from 850 to 420 cm^−1^ were characteristic absorption bands of Al_2_O_3_. For C@ACM, the shoulder peaks at 1707 cm^−1^ and 1743 cm^−1^ could have originated from the stretching vibration of C=O in different chemical environments [56]. The peak at 1625 cm^−1^ was assigned to the C=C stretching vibrations [57]. Additionally, the increased peak at 1087 cm^−1^ could be attributed to the C-O stretching vibration, indicating the formation of the ester group. The intensity of the peak at around 3435 cm^−1^ was decreased due to the conspicuous decrease of -OH on the carbon layer.

The XPS analysis was performed to further investigate the chemical environment of the membranes. As shown in the O 1s spectrum (Figure 4d), the content of AlOOH of C@ACM decreased significantly due to the covered carbon layer. Moreover, new functional groups (O=C-O, C=O, and C-O) appeared on the surface of the C@ACM [57] to adjust the surface properties, which may have had a particularly important influence on the separation processes. The XPS analysis results (Table 2), well consistent with the EDS results of the hydrothermal precipitate, indicated that the surface carbon element content increased while the oxygen element content decreased from 49.56% to 22.50%. The carbon layer was successfully formed during the hydrothermal process. Figure 4d indicates that more functional groups with high C/O ratios (C-O-C, C=O) than the groups with low C/O ratios (O=C-OH) were on the carbon layer.

### 3.2. Acid Resistance Properties

**Figure 5:**
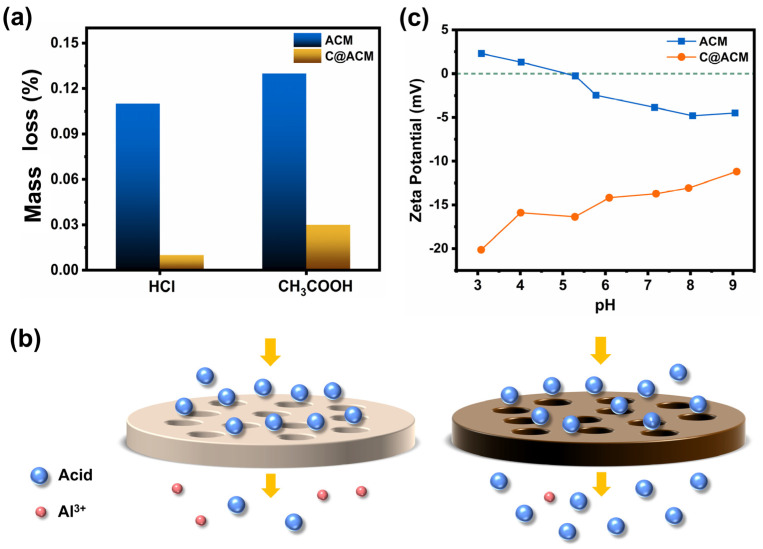
Acid resistance test: (**a**) mass loss of ACM and C@ACM after 24-h immersion in HCl and CH_3_COOH solution (pH = 3.50) under 80 °C; (**b**) Schematic diagram of the acid permeation process; (**c**) ζ potential of the membrane surface.

The lower mass loss reflected the improvement in the membrane acid resistance. As shown in Figure 5, the C@ACM lost mass by 0.01% and 0.03% in the hydrochloric acid solution and acetic acid solution, respectively, which was superior to the 0.25% weight loss reported in the literature [58]. After modification, the membrane mass loss was greatly decreased by 90.91% and 76.92%, which may be attributed to the protection of the carbon layer.

The acid permeation process is simply described in Figure 5b. The dealumination (6H^+^+Al_2_O_3_→3H_2_O+2Al^3+^) was relieved and the acid permeation increased on C@ACM. The charge and the electronegativity of functional groups on the carbon layer may have been due to two key factors. Owing to the dealumination, Al^3+^ was in the solution; thus, the zeta potential (Figure 5c) of ACM at pH = 3.09 was positive (2.30), which is consistent with the literature [59,60]. This method of aluminum loss was essentially related to electrostatic interaction, with which hydrogen ions combined with oxygen anion after getting into the crystal lattice of α-Al_2_O_3_ followed by Al^3+^ getting out of the lattice without the binding of the oxygen anions. Conversely, the zeta potential turned negative after modification. This could be attributed to the introduction of a large number of O-containing functional groups in the carbon layer. As a result, the carbon layer acted as a protective layer to alleviate the dealumination for the negatively charged oxygen atoms on molecules A and B (Figure 2). The Milluiken charges calculated by Gaussian 09W software verified this (Table 3). C@ACM exhibited dominant electrostatic repulsion against negatively charged acid radical ions. The hydrogen ions tended to permeate to the other side of the membrane with acid ions rather than being consumed due to the synergistic effect of the electrostatic equilibrium and the carbon layer blocking access to the crystal lattice.

Moreover, the electronegativity of the surface groups was another reason for the different penetration. The acid may have been hydrogen-bonded to the hydroxyl group on the ACM surface and thus been adsorbed to the membrane surface, resulting in low penetration. After being coated with the carbon layer, many functional groups such as C=O and C-O-C existed on the C@ACM surface. According to the principle of Sanderson’s electronegativity equalization, the electronegativity values of the functional groups with lower oxygen content (C=O, C-O-C, and C-O-H) on C@ACM were between 2.45 and 2.80, which were smaller than that of -OH (3.08) on ACM. The groups with small electronegativity weakened the hydrogen bonding with the acid, and therefore reduced the acid depletion on C@ACM.

### 3.3. Adsorption Kinetics and Thermodynamics Fitting

**Figure 6:**
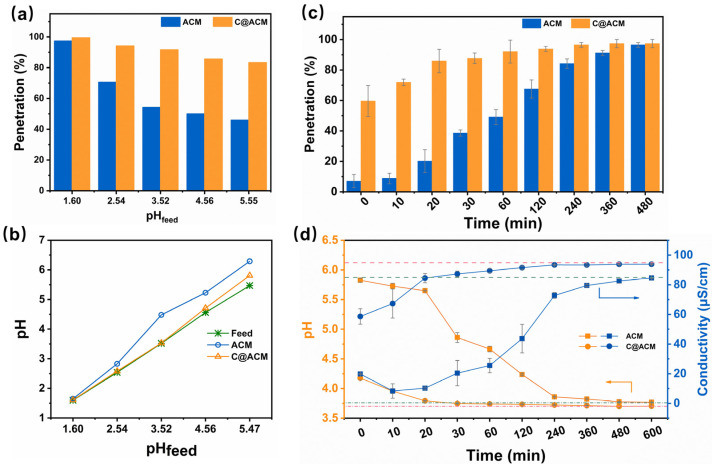
The permeating behavior of HCl through ACM and C@ACM: (**a**) penetration of HCl with different pH; (**b**) pH values of permeation; (**c**) Penetration of HCl; (**d**) pH values, and conductivity of permeation at different times. (The filtration experiments in a and b were ended until the volume of permeate reached 60 mL. The pH values of the feed that permeated through ACM (green dot-and-dash line) and C@ACM (pink dot and dash line) were 3.76 and 3.70, respectively. The conductivity of the feed through ACM (the green dotted line) was 84.9 μS·cm^−1^ and that through C@ACM (the pink dotted line) was 94.9 μS·cm^−1^).

As shown in Figure 6a,b, the pH of the permeation treated by the ACM increased obviously. It was speculated that hydrogen ions were consumed for dealumination during the membrane process. The acid penetration was higher on C@ACM than ACM, and the difference increased with increasing the pH value. Moreover, the acid penetration was related to the permeating time (Figure 6c,d). The acid depletion was more obvious in the early stage during the membrane process, especially on the ACM.

It is worth mentioning that the conductivity (ACM) was higher at the initial time. This might have been due to the dealumination of alumina in an acid solution, resulting in the release of aluminum ions. The retention of the acid in the ACM permeation did not reach 80% until 6 h later. This indicates that the acid depletion on the ACM could not be ignored before reaching the adsorption equilibrium, which was also reflected in the changes in pH value and conductivity. This is because if acid-rich TCM extract solutions or juices were treated with ACM, a large number of acid components would be lost, resulting in the reduction of active ingredients and a change in flavor. This means that at least 6 h should be spent pre-saturating the ACM before each application for filtration to minimize the loss of acid, which would lead to a lot of time being wasted. Notably, C@ACM exhibited less acid depletion during the whole process. The acid content in the C@ACM permeation was eight times more than that in the ACM permeation within the first 10 min. Moreover, the time taken to reach 80% penetration was drastically reduced to 20 min on C@ACM. The C@ACM showed a superior performance since it shortened the time for equilibrium and enhanced the efficiency of the acid solution filtration. These results revealed that the carbon layer could alleviate the loss of the acid components, indicating an improvement in the acid resistance of the membrane.

**Figure 7:**
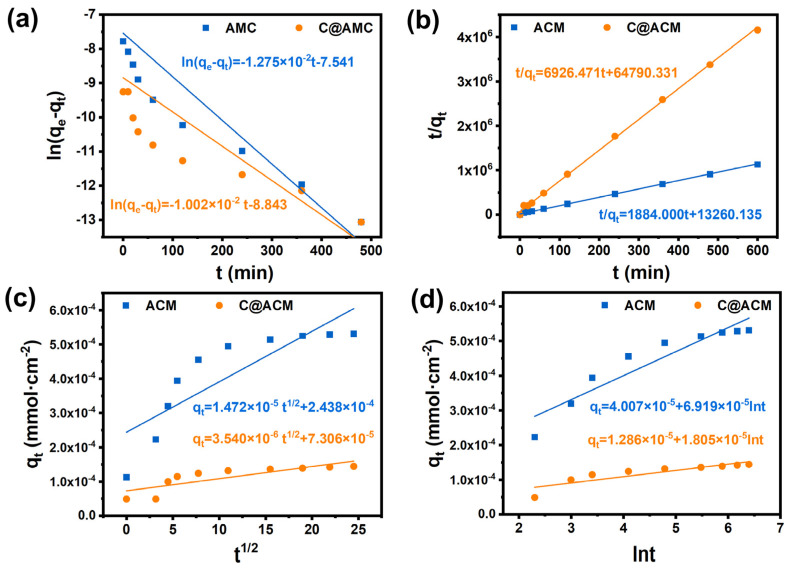
Adsorption kinetics of ACM and C@ACM: (**a**) pseudo-first order; (**b**) pseudo-second order; (**c**) the Weber and Morris model; (**d**) the Elovich model.

The kinetic adsorption equilibrium fittings were carried out to explore the adsorption mechanism to describe the consumption process. The pseudo-first-order, the pseudo-second-order, the Weber and Morris model, and the Elovich model were employed to fit the experimental data (Figure 7 and Table 4). According to Table 4, the pseudo-second-order model showed the best fitting for both ACM and C@ACM with the R^2^ reaching 0.9994 and 0.9992, respectively. The adsorption rate constant K_2_ of C@ACM (6926.471) was much higher than that of ACM (11,884.000), revealing the faster adsorption equilibrium for C@ACM.

**Figure 8:**
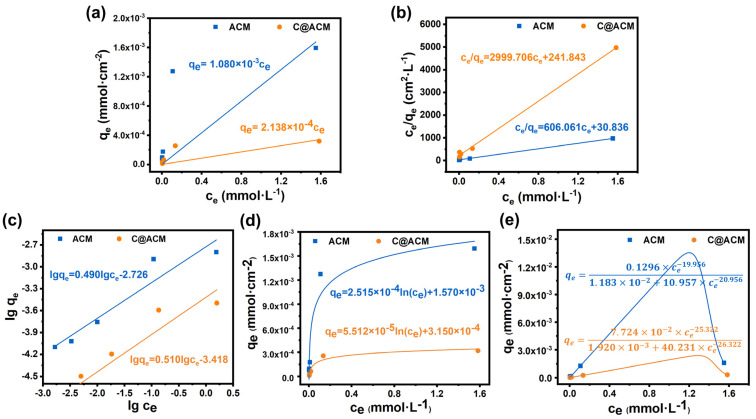
Adsorption isotherms of ACM and C@ACM: (**a**) Henry; (**b**) Langmuir; (**c**) Freundlich; (**d**) Tempkin; (**e**) Radke–Prausnitz models.

The adsorption isotherms are illustrated in Figure 8, and the values of R^2^ are tabulated in Table 5. According to the R^2^ values (Table 5), the equilibrium data of ACM and C@ACM can be well explained by the Langmuir model, indicating the monolayer adsorption on the membranes. The decreased parameter q_max_ and K_L_ revealed that the adsorption capacity of acid by C@ACM was weakened.

### 3.4. Membrane Performance

#### 3.4.1. Penetration of Acid Components

**Figure 9:**
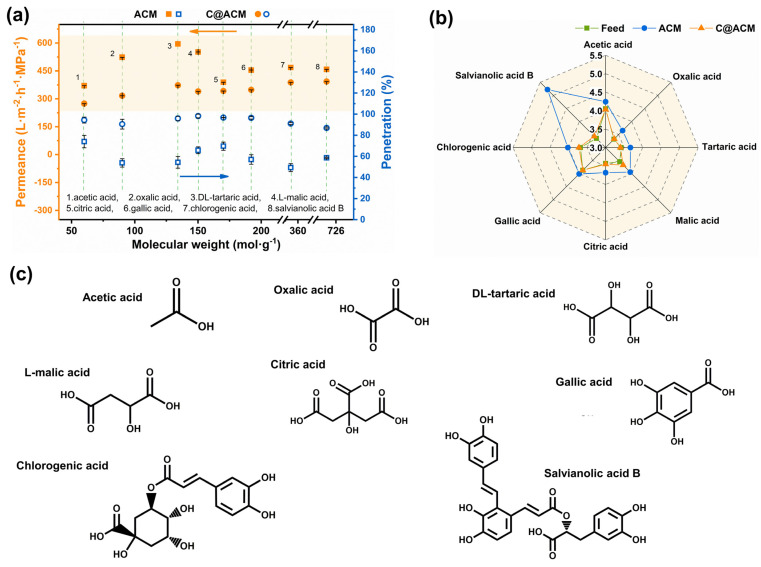
Permeation results of the nine acids through ACM and C@ACM: (**a**) permeance and penetration; (**b**) pH values of feed and permeation; (**c**) chemical structures of the eight acids.

Several kinds of fatty acids and aromatic acids, which widely exist in plants, were employed to explore the practicability of the ceramic membrane in acid solutions. As shown in Figure 9a, for C@ACM, the acid penetration was kept at a high level with slightly decreased fluxes. For example, the transmittance rate of hydrochloric acid was improved from 41.02% (ACM) to 95.73% (C@ACM). This indicated the C@ACM was more suitable for the acid-rich solution to retain the acid components in the permeate.

#### 3.4.2. Performance of Acidic Extract Solutions

**Figure 10:**
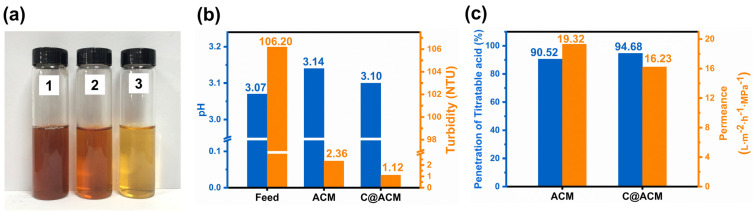
Properties of hawthorn extract after membrane treatment: (**a**) The photos of hawthorn extract: 1. feed, 2. ACM permeate, 3. C@ACM permeate; (**b**) pH and turbidity of the feed and permeates; (**c**) The flux and penetration of titratable acid.

Hawthorn is rich in varieties of organic acids such as tartaric acid, citric acid, and malic acid, et al. Therefore, the hawthorn extract solution was chosen as a characteristic extract to explore the practical application potential of the membranes. As shown in Figure 10a, the color became lighter and cleaner after the C@ACM treatment, indicating a better decolorization effect than the ACM. Moreover, the permeate exhibited a higher clarity after the C@ACM treatment, as the turbidity decreased from 106.20 to 1.12 (Figure 10b).

The permeance of the C@ACM decreased slightly due to the narrowed pore size compared with the ACM (Figure 10c). In general, the C@ACM was more suitable for the application of a practical acid system due to the lower acid component loss.

The pH value closer to the original solution (Figure 10b) and the higher penetration of titratable acid (Figure 10c) indicated that the C@ACM exhibited a better acid solution treatment ability than the ACM.

### 3.5. Durability Test and Recycling

**Figure 11:**
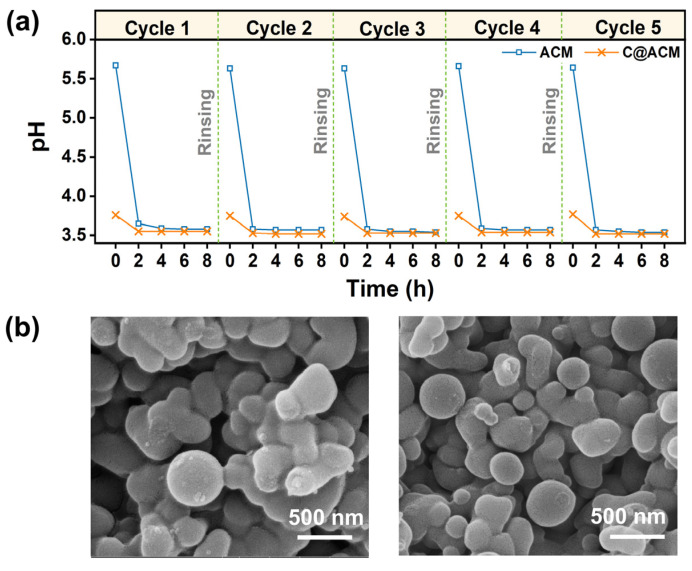
(**a**) the cyclic performance of ACM and C@ACM; (**b**) The SEM images of C@ACM before (the left one) and after (the right one) permeating the hydrochloric acid solution under 0.2 MPa.

The cyclic performance was tested by repeated rinsing and testing to evaluate durability. As shown in Figure 11a, both ACM and C@ACM showed the same trend for acid depletion in every cycle. This indicates the reversible physical adsorption to acid, which was consistent with the results of the adsorption isotherm analysis. The ACM always caused conspicuous acid loss in the first two hours, while the C@ACM could maintain its advantages in each use. Moreover, the morphology (Figure 11b) of the carbon layer and the carbon spheres on C@ACM were kept intact after permeating the acid solution. This indicated that the C@ACM would not be corroded by the acid solution. The chemical oxygen demand (COD) value of the acid solution permeate was the same as that of the ultrapure water, which was further consistent with the SEM results. Simultaneously, these results also revealed that the C@AM was stable enough to withstand repeated use for a long time.

**Figure 12:**
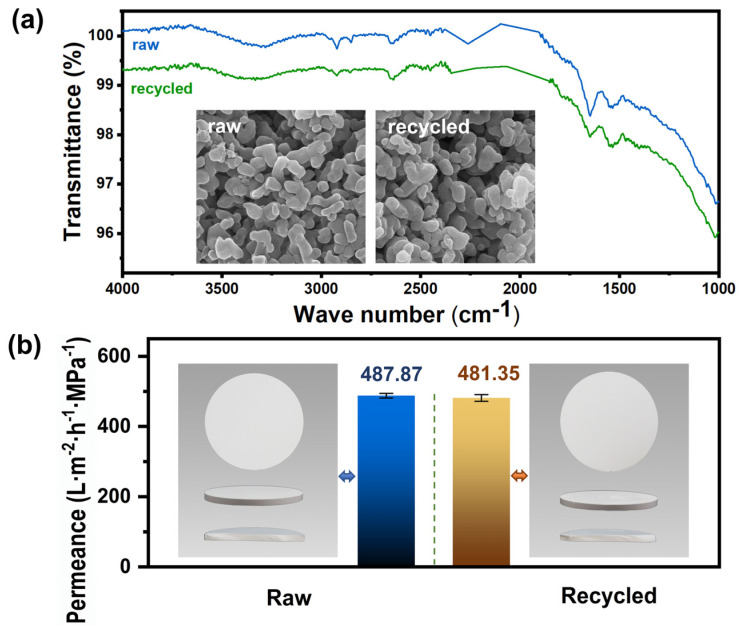
The comparison of the raw and the recycled ACM: (**a**) FT-IR spectra and SEM images; (**b**) the photograph and pure water permeance.

As shown in Figure 12a, the FT-IR results showed similar chemical functional groups on the raw and the recycled ACM. Moreover, the morphology of the recycled ACM after calcination almost had no difference from that of the raw ACM. The EDS mapping result (Figure S7) showed that the carbon element proportion of the recycled ACM (0.26%) was almost the same as that of the raw ACM (0.71%). This indicated that the carbon layer was completely removed after calcination at 550 °C. Additionally, the water flux recovery of the recycled membrane was up to 98.66% (Figure 12b). Thus, the membrane can be recycled by high-temperature heating.

## 4. Conclusions

Acid resistance is an important factor affecting the performance of the ceramic membrane with application in acid solution treatment. This work reported an acid-resistant alumina ceramic membrane prepared with a facile one-step in situ hydrothermal method and designed for the treatment of acidic solutions. The outer carbon shell acted as a protective layer for the membrane, making it more resistant to acid. After modification, the mass losses of membranes treated with hydrochloric acid solution and acetic acid solution decreased by 90.91% and 76.92%, respectively. Adsorption models were employed to describe the acid depletion processes of the membranes. The pseudo-second order adsorption kinetics enabled the faster equilibrium for C@ACM. Moreover, the isotherm adsorption model revealed the decreased adsorption capacity of acid with the carbon coating. The C@ACM enabled the preservation of various common acid substances as well as the active acid components in hawthorn extract to maintain the flavor with a high flux. Moreover, the excellent durability and recyclability demonstrate that C@ACM constitutes a new candidate for the treatment of acidic solutions.

## Figures and Tables

**Table 1:** The atomic ratio of Al, O, and C from ACM, C@ACM, and hydrothermal precipitate tested by EDS mapping.

Material	Position	Al ^a^	O ^b^	C ^c^	Total
ACM	Surface	43.23%	53.99%	2.78%	100%
	Cross-section	44.54%	54.64%	0.82%	100%
C@ACM	Surface	36.60%	47.15%	16.25%	100%
	Cross-section	34.56%	55.84%	9.62%	100%
Hydrothermal precipitate	Surface	0	24.72%	75.28%	100%

^a,b,c^ Data determined by EDS mapping with SEM (QUANTA FEG 250, USA).

**Table 2:** The atomic ratio of the ceramic membrane surface was tested by XPS.

Membranes	Al 2p	O 1s	C 1s
ACM	29.09%	49.56%	21.35%
C@ACM	1.04%	22.50%	76.46%

**Table 3:** The calculated Milluiken charge of the O atom from molecular A and B.

Origin	Number	Milluiken Charge ^a^
Molecular A	O19	−0.6405
	O22	−0.4108
	O23	−0.5408
	O24	−0.5318
Molecular B	O20	−0.5611
	O35	−0.3591
	O36	−0.4079
	O37	−0.5479
	O45	−0.3839

^a^ Data calculated by Gaussian 09W by importing the compound chemical structures into the GaussView software. Before being imported, the chemical structures were drawn by ChemDraw 18.0 software followed by structural optimization and energy minimization by Chem3D software.

**Table 4:** Kinetic parameters for H+ adsorption.

Adsorption Isotherm Model	Parameters	Adsorption	
		ACM	C@ACM
Pseudo first order	K_1_	1.275 × 10^−2^	1.002 × 10^−2^
	R^2^	0.9939	0.9929
Pseudo second order	K_2_	1884.000	6926.471
	R^2^	0.9994	0.9992
The Weber and Morris model	K_ip_	1.472 × 10^−5^	3.540 × 10^−6^
	C	2.438 × 10^−4^	7.306 × 10^−5^
	R^2^	0.6931	0.6435
Elovich model	α	1.235 × 10^−4^	3.681 × 10^−5^
	β	1.445 × 10^4^	5.541 × 10^4^
	R^2^	0.8710	0.7532

**Table 5:** Isotherm parameters for H^+^ adsorption.

Adsorption Isotherm Model	Parameters	Adsorption	
		ACM	C@ACM
Henry	H	1.080 × 10^−3^	2.138 × 10^−4^
	R^2^	0.5878	0.5913
Langmuir	q_max_	1.650 × 10^−3^	3.334 × 10^−4^
	K_L_	19.645	13.962 × 10^−2^
	R^2^	0.9987	0.9975
Freundlich	n	2.040	1.961
	K_F_	1.878 × 10^−3^	3.821 × 10^−4^
	R^2^	0.8894	0.80372
Tempkin	A	1.570 × 10^−3^	3.150 × 10^−4^
	B	2.515 × 10^−4^	5.512 × 10^−5^
	R^2^	0.9074	0.9311
Radke–Prausnitz	a	1.183 × 10^−2^	1.920 × 10^−3^
	b	10.957	40.231
	β	−19.956	−25.322
	R^2^	0.9906	0.9659

## Data Availability

Not applicable.

## Supplementary Materials

The following supporting information can be downloaded at: https://www.mdpi.com/article/10.3390/membranes12121246/s1, Figure S1: Cross-section of ACM: (a) Diagram; (b) SEM image; Figure S2: Pure water permeance of ACM and C@ACM; Figure S3: XRD patterns of ACM and C@ACM; Figure S4: XPS spectra of the hydrothermal precipitate; Figure S5: Water contact angle: (a) ACM and (b) C@ACM; Figure S6: Dynamic transmittance rate and pH of chlorogenic acid solution permeating through the ACM and C@ACM; Figure S7: The EDS results of the membranes: (a) the raw ACM, (b) the C@ACM, (c) the recycled ACM; Table S1: Pore size of the ceramic membranes; Table S2: The parameters of the membranes; Table S3: The coefficient f and K in works listed in Table S2. Refs. [61,62,63,64,65,66,67] are cited on the supplementary materials.

## References

[1] Ojalvo C., Fuentes M.J., Zhang W.J., Guiberteau F., Candelario V.M., Ortiz A.L. (2022). Fabrication of B4C ultrafiltration membranes on SiC supports. J. Eur. Ceram. Soc..

[2] Alftessi S.A., Othman M.H.D., Adam M.R., Farag T.M., Ismail A.F., Rahman M.A., Jaafar J., Habib M.A., Raji Y.O., Hubadillah S.K. (2021). Novel silica sand hollow fiber ceramic membrane for oily wastewater treatment. J. Environ. Chem. Eng..

[3] Mao X., Zhao L., Zhang K., Wang Y.Y., Ding B. (2022). Highly flexible ceramic nanofibrous membranes for superior thermal insulation and fire retardancy. Nano Res..

[4] Liu F., Yao H., Sun S.B., Tao W.J., Wei T., Sun P.Z. (2020). Photo-fenton activation mechanism and antifouling performance of an FeOCl-coated ceramic membrane. Chem. Eng. J..

[5] Gu Q.L., Albert Ng T.C., Zhang L., Lyn Z.Y., Zhang Z.X., Yong H., Wang J. (2020). Interfacial diffusion assisted chemical deposition (ID-CD) for confined surface modification of alumina microfiltration membranes toward high flux and anti-fouling. Sep. Purif. Technol..

[6] Liu W., Yang G., Huang M.P., Liang J.W., Zeng B.B., Fu C., Wu H.D. (2020). Ultrarobust and biomimetic hierarchically macroporous ceramic membrane for oil-water separation templated by emulsion-assisted self-assembly method. ACS Appl. Mater. Inter..

[7] Asif M.B., Zhang Z.H. (2021). Ceramic membrane technology for water and wastewater treatment: A critical review of performance, full-scale applications, membrane fouling and prospects. Chem. Eng. J..

[8] Zhong W.W., Zheng D.Y., Zou H.H., Zhuo Z.W., Guo L.W. (2021). A novel approach for evaluating the concentrations of Indicative components in liquid and solid in the pharmaceutical process of TCM manufacturing using membrane-based clarification--example given in the water extracts of eucommiae folium. Chin. Tradit. Herb. Drugs..

[9] Li C., Lu Z.D., Ao X.W., Sun W.J., Huang X. (2022). Degradation kinetics and removal efficiencies of pharmaceuticals by photocatalytic ceramic membranes using ultraviolet light-emitting diodes. Chem. Eng. J..

[10] Aloulou W., Aloulou H., Attia A., Chakraborty S., Amar R.B. (2022). Treatment of tuna cooking juice via ceramic ultrafiltration membrane: Optimization using response surface methodology. Membranes.

[11] Mo J.H., Li X.H., Yang Z.F. (2022). Dissecting the structure-property relationship of ceramic membrane with asymmetric multilayer structures for maximizing permselectivity. Water Res..

[12] Zou D., Chen X.F., Drioli E., Qiu M.H., Fan Y.Q. (2019). Facile mixing process to fabricate fly ash-enhanced alumina-based membrane supports for industrial microfiltration applications. Ind. Eng. Chem. Res..

[13] Hang Y.T., Liu G.P., Huang K., Jin W.Q. (2015). Mechanical properties and interfacial adhesion of composite membranes probed by in-situ nano-indentation/scratch technique. J. Mem. Sci..

[14] Lu M., Hu M.Z. (2020). Novel porous ceramic tube-supported polymer layer membranes for acetic acid/water separation by pervaporation dewatering. Sep. Purif. Technol..

[15] Bukhari S.Z.A., Ha J.H., Lee J.M., Song I.H. (2017). Fabrication and optimization of a clay-bonded SiC flat tubular membrane support for microfiltration applications. Ceram. Int..

[16] Ivanets A.I., Azarova T.A., Agabekov V.E., Azarov S.M., Batsukg C., Batsuren D., Prozorovich V.G., Pat’to A.A. (2016). Effect of phase composition of natural quartz raw material on characterization of microfiltration ceramic membranes. Ceram. Int..

[17] Hoffman R., Pippardt U., Kriegel R. (2019). Impact of sintering temperature on permeation and long-term development of support structure and stability for asymmetric oxygen transporting BSCF membranes. J. Mem. Sci..

[18] Guo H.L., Zhao S.F., Wu X.X., Qi H. (2016). Fabrication and characterization of TiO2/ZrO2 ceramic membranes for nanofiltration. Micropor. Mesopor. Mat..

[19] Luo Z.Y., Han W., Liu K.Q., Ao W.Q., Si K.K. (2019). Influence of bonding phases on properties of in-situ bonded porous SiC membrane supports. Ceramics International. Ceram. Int..

[20] Adam M.R., Othman M.H.D., Kadir S.H.S.A.L., Sokri M.N.M., Tai Z.S., Iwamoto Y., Tanemura M., Honda S., Puteh M.H., Rahman M.A. (2020). Influence of the Natural Zeolite Particle Size Toward the Ammonia Adsorption Activity in Ceramic Hollow Fiber Membrane. Membranes.

[21] Gu Q.L., Albert Ng T.C., Bao Y.P., Yong H., Tan S.C., Wang J. (2020). Developing better ceramic membranes for water and wastewater Treatment: Where microtructure integrates with chemistry and functionalities. Chem. Eng. J..

[22] Chu K.H., Fathizadeh M., Yu M., Flora J.R.V., Jang A., Jang M., Park C.M., Yoo S.S., Her N., Yoon Y. (2017). Evaluation of Removal Mechanisms in a Graphene Oxide-Coated Ceramic Ultrafiltration Membrane for Retention of Natural Organic Matter, Pharmaceuticals, and Inorganic Salts. ACS Appl. Mater. Inter..

[23] Karan S., Jiang Z.W., Livingston A.G. (2015). Sub-10 nm polyamide nanofilms with ultrafast solvent transport for molecular separation. Science..

[24] Priyadarshini A., Tay S.W., Hong L. (2021). Zeolite Composite Membranes with a Nanoporous Fluorinated Carbonaceous Sheath for Organic Solvent Filtration. ACS Appl. Nano Mater..

[25] Justino N.M., Vicentini D.S., Ranjbari K., Bellier M., Nogueira D.J., Matias W.G., Perreault F. (2021). Nanoparticle-templated polyamide membranes for improved biofouling resistance. Environ. Sci-Nano.

[26] Chee D.N.A., Ismail A.F., Aziz F., Amin M.A.M., Abdullah N. (2020). The influence of alumina particle size on the properties and performance of alumina hollow fiber as support membrane for protein separation. Sep. Purif. Technol..

[27] Zhou M., Nabavi M.S., Hedlund J. (2020). Influence of support surface roughness on zeolite membrane quality. Micropor. Mesopor. Mat..

[28] Araya M.V., Oeltze H., Radeva J., Roth A.G., Gobbert C., Pahl R.N., Dahne L., Wiese J. (2022). Operation of Hybrid Membranes for the Removal of Pharmaceuticals and Pollutants from Water and Wastewater. Membranes.

[29] Hammad I., Dornier M., Lebrun M., Maraval I., Poucheret P., Mayer C.D. (2022). Impact of crossflow microfiltration on aroma and sensory profiles of a potential functional citrus-based food. J. Sci. Food Agr..

[30] Alawy R.M.J.A., Abod B.M., Kamar F.H., Nechifor A.C. (2019). Removal of Dyes from Wastewater by Ceramic Membrane. Rev. Chim. Bucharest..

[31] Changmai M., Mondal P., Sinha A.P.B., Biswas P., Sarkar S., Purkait M.K. (2022). Metal removal efficiency of novel LD-slag-incorporated ceramic membrane from steel plant wastewater. Int. J. Environ. An. Ch..

[32] Bian W.J., Wang B.M., Tang W., Zhou M., Jin C.R., Ding H.P., Fan W.W., Dong Y.H., Li J., Ding D. (2022). Revitalizing interface in protonic ceramic cells by acid etch. Nature.

[33] Dang Q., Lin H.P., Fan Z.L., Ma L., Shao Q., Ji Y.J., Zeng F.F., Geng S.Z., Yang S.Z., Kong N.N. (2021). Iridium metallene oxide for acidic oxygen evolution catalysis. Nat. Commun..

[34] Ma J., Du B., He C., Zeng S.H., Hua K.H., Xi X., Luo B.Y., Shui A.Z., Tian W. (2022). Corrosion Resistance Properties of Porous Alumina-Mullite Ceramic Membrane Supports. Adv. Eng. Mater..

[35] Ferdowsi S., Salem A., Salem S. (2019). Spectrophotometrical analysis for fabrication of pH-independent nano-sized gamma-alumina by dealumination of kaolin and precipitation in the presence of surfactant composites. Spectrochim. Acta. A..

[36] Schacht M., Boukis N., Dinjus E. (2000). Corrosion of alumina ceramics in acidic aqueous solutions at high temperatures and pressures. J. Mater. Sci..

[37] Lin G.S., Liu Y.C., Anbarasan R., Naakagawa K., Yoshioka T., Matsuyama H., Tseng H., Tung K.L. (2020). Silica gel-coated silicon carbide layer deposited by atmospheric plasma spraying. J. Taiwan Ins. Chem. E..

[38] Tuci G.L., Liu Y.F., Rossin A., Guo X.Y., Pham C., Giambastiani G., Cuong P. (2021). Porous Silicon Carbide (SiC): A Chance for Improving Catalysts or Just Another Active-Phase Carrier?. Chem. Rev..

[39] Muthu M., Santhanam M. (2018). Effect of reduced graphene oxide, alumina and silica nanoparticles on the deterioration characteristics of Portland cement paste exposed to acidic environment. Cement Concrete Comp..

[40] Chen H.J., Zhang Z.B., Zhong X., Zhuo Z.J., Tian S.L., Fu S.Y., Chen Y., Liu Y.H. (2021). Constructing MoS2/Lignin-derived carbon nanocomposites for highly efficient removal of Cr (VI) from aqueous environment. J. Hazard. Mater..

[41] Wang J.L., Wang S.Z. (2019). Preparation, modification and environmental application of biochar: A review. J. Clean. Prod..

[42] Frenzel L.M., Roland U., Kopinke F.D. (2021). Coating of solid substrates with carbon via hydrothermal carbonization. Mater. Lett..

[43] Liu J., Li S.J., Wang C., Deng C.M., Mao J., Tan X., Li W., Zhang P., Wang Q.W. (2022). Self-lubricating design strategy for thermally sprayed ceramic coatings by in-situ synthesis of carbon spheres. Surf. Coat. Tech..

[44] Hofmann S., Koch D., Baranger E., Lamon J. (2015). Predicting the mechanical behavior of carbon fiber reinforced silicon carbide with interlaminar manufacturing defects. Clin. Neuroradiol..

[45] Feng Q.H., Lin P.P., Ma G.L., Lin T.S., He P., Long W.M., Zhang Q.G. (2021). Design of multi-layered architecture in dissimilar ceramic/metal joints with reinforcements clustering away from both substrates. Mater. Design.

[46] Feng Q.H., Lin P.P., Lin T.S., He P., Liu Y., Long W.M., Li J. (2021). Controllable distribution of reinforcements for reducing the strain energy in dissimilar ceramic/metal joints. J. Eur. Ceram. Soc..

[47] Ouyang H.B., Li G.B., Li C.Y., Huang J.F., Fei J., Lu J. (2018). Microstructure and ablation properties of C/C-Zr-Si-O composites prepared by carbothermal reduction of hydrothermal co-deposited oxides. Mater. Design.

[48] Zhu Y., Zhang Q., Meng X.L., Yan L.S., Cui H. (2020). Adhesive joint properties of advanced carbon/ceramic composite and tungsten-copper alloy for the hybrid rocket nozzle. Int. J. Adhes. Adhes..

[49] Ismail N.H., Salleh W.N.W., Sazali N., Ismail A.F. (2017). Effect of intermediate layer on gas separation performance of disk supported carbon membrane. Sep. Sci. Technol..

[50] Wang C., Ling L., Huang Y., Yao Y.G., Song Q. (2015). Decoration of porous ceramic substrate with pencil for enhanced gas separation performance of carbon membrane. Carbon.

[51] Chen Y.Q., Zhang X., Chen W., Yang H.P., Chen H.P. (2017). The structure evolution of biochar from biomass pyrolysis and its correlation with gas pollutant adsorption performance. Bioresource Technol..

[52] Zhang C.P., Xia Q., Han L.F., Zhao Y.L., Huang N., Ren Q.X., Zhang X., Ru H.Q. (2022). Fabrication of carbon-coated boron carbide particle and its role in the reaction bonding of boron carbide by silicon infiltration. J. Eur. Ceram. Soc..

[53] Xu R.L., Bian D., Aradhyula T.V., Chavali M., Zhao Y.W. (2019). Preparation and corrosion behavior studies of chemically bonded phosphate ceramic coating reinforced with modified multi-walled carbon nanotubes (MWCNTs). Ann. Am. Thorac. Soc..

[54] Zhang Y.H., Wang N.N., Sun C.H., Lu Z.X., Xue P., Tang B., Bai Z.C., Dou S.X. (2018). 3D spongy CoS2 nanoparticles/carbon composite as high-performance anode material for lithium/sodium ion batteries. Chem. Eng. J..

[55] Wang X.H., Jiang C.L., Hou B.X., Wang Y.Y., Hao C., Wu J.B. (2018). Carbon composite lignin-based adsorbents for the adsorption of dyes. Chemosphere.

[56] Liu Y.J., Ren D.Z., Song Z.Y., Wan X.Y., Zhang C.T., Jin F.M., Huo Z.B. (2018). A novel method to prepare a magnetic carbon-based adsorbent with sugar-containing water as the carbon source and DETA as the modifying reagent. Environ. Sci. Pollut. R..

[57] Mujmle R.B., Chung W.J., Kim H. (2020). Chemical fixation of carbon dioxide catalyzed via hydroxyl and carboxyl-rich glucose carbonaceous material as a heterogeneous catalyst. Chem. Eng. J..

[58] Cui K.J., Li P., Zhang R., Cao B. (2020). Preparation of pervaporation membranes by interfacial polymerization for acid wastewater purification. Chem. Eng. Res. Des..

[59] Webber J., Zorzi J.E., Perottoni C.A., Silva S.M.E., Cruz R. (2016). Identification of α-Al_2_O_3_ surface sites and their role in the adsorption of stearic acid. J. Mater. Sci..

[60] Bowers A., Huang C.P. (1985). Adsorption characteristics of polyacetic amino acids onto hydrous γ-Al_2_O_3_. J. Colloid Inter. Sci..

[61] Fan Y., Zhou Y., Feng Y., Wang P., Li X.Y., Shih K.M. (2020). Fabrication of reactive flat-sheet ceramic membranes for oxidative degradation of ofloxacin by peroxymonosulfate. J. Mem. Sci..

[62] Li X.B., Zhou Z.H., Chen L., Kong C.L., Du H.B. (2019). Design and Synthesis of Acid-resistant Zeolite T/NaY Composite Membrane for Water/Ethanol Separation. B. Korean Chem. Soc..

[63] Zhang S.B., Zhong J.X., Zhao S.K., Suo N., Liu G.C., Xie K., Wang F.C., Liu Y.Y., Ju W.P. (2021). Operational optimization control and membrane fouling mechanism analysis of ceramic membrane treating secondary treated effluent. Chin. J. Environ. Eng..

[64] Qin H., Guo W.M., Gao P.Z., Xiao H.N. (2020). Spheroidization of low-cost alumina powders for the preparation of high-flux flat-sheet ceramic membranes. Ceram. Int..

[65] Song I.H., Bae B.S., Ha J.H., Lee J.M. (2017). Effect of hydraulic pressure on alumina coating on pore characteristics of flat-sheet ceramic membrane. Ceram. Int..

[66] Majumder A., Gupta A.K., Ghosal P.S., Varma M. (2021). A review on hospital wastewater treatment: A special emphasis on occurrence and removal of pharmaceutically active compounds, resistant microorganisms, and SARS-CoV-2. J. Environ. Eng..

[67] Ji B., Zhu L., Wang S.L., Liu Y. (2021). Temperature-effect on the performance of non-aerated microalgal bacterial granular sludge process in municipal wastewater treatment. J. Environ. Manag..

