# Supplementary material for: Core-Shell Structured Carbon@Al2O3 Membrane with Enhanced Acid Resistance for Acid Solution Treatment
Source: Membranes (Basel). 2022 Dec 9;12(12):1246. doi: 10.3390/membranes12121246 (PMC9784977; doi:10.3390/membranes12121246)
Supplement: Supplementary file 1 [file membranes-12-01246-s001.zip › membranes-2040190-supplementary.pdf]

Supporting information

Core-shell Structured Carbon@Al_2O_3 Membrane with Enhanced Acid Resistance for Acid Solution Treatment

Qianlian Wu [1,2], Huimiao Zhang [1,2], Yi Zhou [1,2], Zhishu Tang [3,4], Bo Li [1,2], Tingming Fu [1,2], Yue Zhang [1,2,*], and Huaxu Zhu [1,2,*]

[1] Jiangsu Collaborative Innovation Center of Chinese Medicinal Resources Industrialization, Nanjing University of Chinese Medicine, Nanjing 210023, China
[2] Jiangsu Botanical Medicine Refinement Engineering Research Center, Nanjing University of Chinese Medicine, Nanjing 210023, China
[3] State Key Laboratory of Research & Development of Characteristic Qin Medicine Resources, Shaanxi University of Chinese Medicine, Xianyang 712038, China
[4] China Academy of Chinese Medical Sciences, Beijing 100700, China
* Correspondence: zhyue@njucm.edu.cn (Y.Z.); Zhuhx@njucm.edu.cn (H.Z.)

1. Cross-section of ACM

Figure S1 indicated that the ACM exhibited a typical symmetric porous structure.

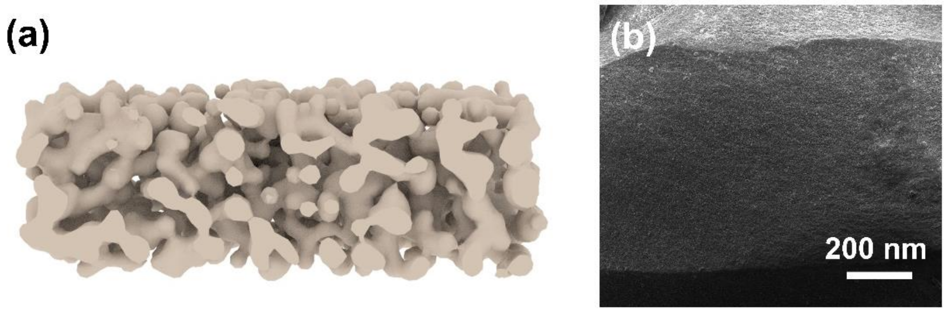

Figure S1. Cross-section of ACM: (**a**) Diagram; (**b**) SEM image.

2. Pure water permeance of ACM and C@ACM

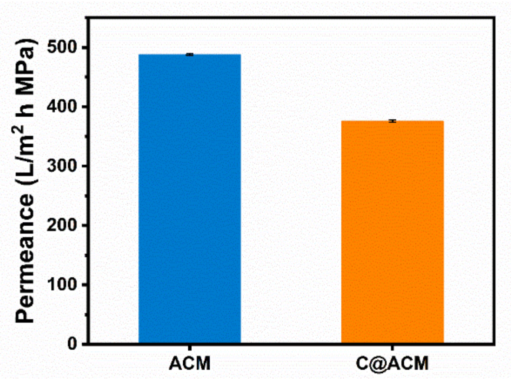

Figure S2. Pure water permeance of ACM and C@ACM.

3. XRD patterns of ACM and C@ACM

The crystalline structures of ACM and C@ACM were illustrated by XRD spectra (Figure S3). The characteristic peaks of ACM and C@ACM were both in accordance with α-Al_2O_3 (JCPDS File NO.10-0173). It indicated the crystal form did not change after modification.

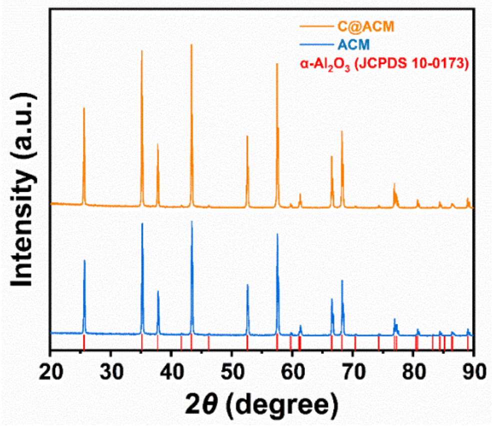

Figure S3. XRD patterns of ACM and C@ACM.

4. XPS Spectra of the hydrothermal precipitate

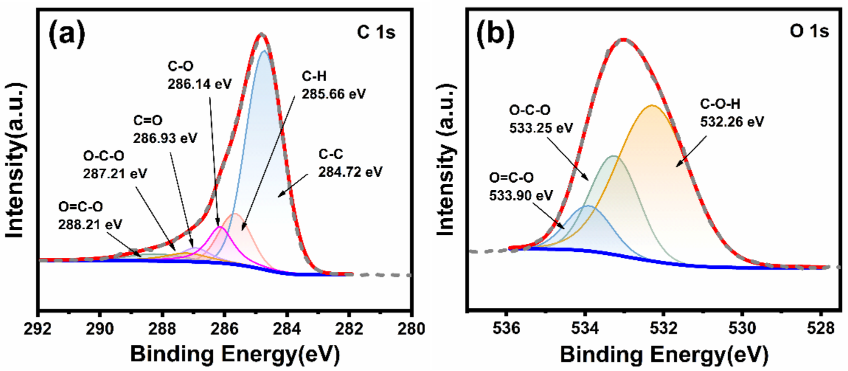

Figure S4. XPS spectra of hydrothermal precipitate: (a) C 1s; (b) O 1s.

5. Parameters of membrane pore size

Table S1. Pore size of the ceramic membranes.

Materials	Average pore size (nm)	Porosity (%)
ACM	148.93	51.05%
C@ACM	107.48	42.38%

6. Water contact angels

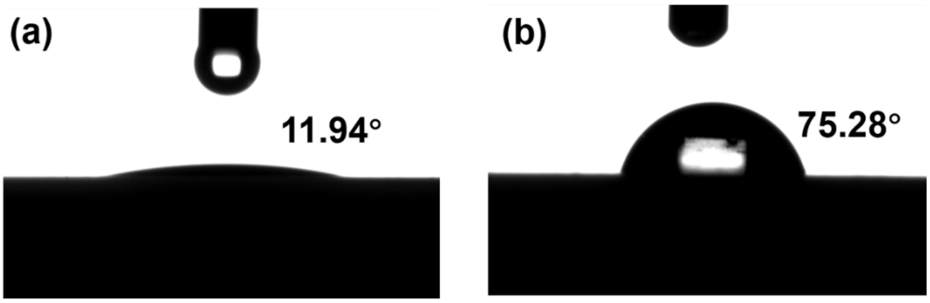

Figure S5. Water contact angle: (**a**) ACM and (**b**) C@ACM.

7. Long time dynamic adsorption experiment of chlorogenic acid

To further confirm the weakened adsorption of acid components on C@ACM, chlorogenic acid solution further was taken as the example. The dynamic transmittance rate of chlorogenic acid through the ACM and C@ACM was detected, and the pH of permeation at different times

was studied. As shown in Figure S6, the transmittance rate through the ACM was less than 10 % in the first 0.5 h, and then increased sharply at 1 h. At the same time, the pH of the permeate in the first 1 h was obviously higher than the feed. It didn't recover to the initial level until 8 h. While the transmittance rate of chlorogenic acid through the C@ACM kept over 95 %. The pH of the permeate was close to the pH of the feed throughout the whole process. The results indicated the chlorogenic acid was adsorbed on the ACM quickly, especially in the first one hour. For C@ACM, the carbon shell obviously decreased the adsorption.

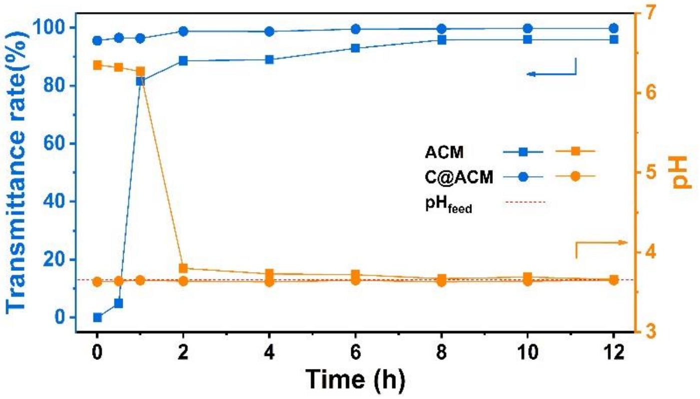

Figure S6. Dynamic transmittance rate and pH of chlorogenic acid solution permeating through the ACM and C@ACM.

8. The parameters of the flat-sheet ceramic membranes

The fluxes as well as the other parameters of the flat-sheet ceramic membranes in this work and other literatures were listed in the following table.

Table S2. The parameters of the membranes.

Name	Flow direction	Average pore size (nm)	Porosity (%)	Thickness	Flux (L/m2hMPa)	Solution	Source
C@ACM	dead-end	107	42	2 mm	376	pure water	this work
Al_2O_3	dead-end	80	-	1.65 mm	823	pure	[61]

substrates						water	
CFCM	dead-end	45	-	~1.65 mm	555	pure water	[61]
T/NaY	cross-flow	200	~50	8 μm	4350	pure water	[62]
Al_2O_3	cross-flow	100	-	-	3384	pure water	[63]
Al_2O_3	cross-flow	120	-	23.5 μm	8500	pure water	[64]
Alumina membrane	cross-flow	130 (top layer); 11000 (support layer)	-	consist of the top layer (40 μm) with a support layer (52 mm)	4500	pure water	[65]

The flux positively correlated to the pore size, and negatively correlated to the membrane thickness. Here, the coefficient f was defined as the ratio of the thickness l (mm) and the pore size d (nm), which can be represented as Eq (1) and (2):

$$f = d/l \quad (1)$$

$$f_n = \Sigma f_i = \Sigma(d_i/l_i) = d_1/l_1 + \dots + d_n/l_n \quad (2)$$

The coefficient K was defined to describe the permeability of the specific materials and flow direction, which was written as Eq (3): $F = Kf$, where the F was the Flux.

Table S3. The coefficient f and K in works listed in Table S2.

Sources	f	K
This work	53.5	7.03
[61]	48.48	18.10
[62]	27.27	20.35
[63]	25000	0.174

[64]	5106.38	1.664
[65]	536.54	8.387

As shown in the Table S3, the K value of this work was lower than those in literature 1, 2 and 5, indicating the higher permeability of C@ACM.

9. Measurement of the chemical oxygen demand (COD) of the C@ACM permeate

The chemical oxygen demand (COD) is an important indicator to measure the content of organic matter in water [66,67]. To assess the combination of the carbon layer as well as the carbon spheres and the C@ACM, the rapid digested spectrophotometry method applied to detect COD of the permeate. The permeate of the hydrochloric acid solution (pH 3.5) through the C@ACM was collected. The C@ACM had been thoroughly cleaned before use. The Deionized water was set as the blank sample. The multifunctional water quality tester (LY-3D, Lvyu, China) equipped with the oxidizing agent ($K_2Cr_2O_7$) and the catalytic agent (Ag_2SO_4) was employed. The measurement method was followed as the instruction of the multifunctional water quality tester. Briefly, 3 mL of the sample was taken into a test tube, and 1 mL of the oxidizing agent was added, followed by 5 mL of the catalytic agent immediately. The COD analyzer was adjusted to low concentration range mode (≤120 mg·L-1) and preheated to 165℃ . The test tube was put in it for digestion for 10 min. After cooling to room temperature, 3 mL of water was added into the test tube. After mixing, the COD value of the sample was determined. The deionized water was set as the first sample, and the COD value was calibrated to zero. Then, the acid permeate solution of C@ACM was determined. As a result, the COD value of the acid permeate solution was zero. This indicates that there was almost no carbon layer or carbon spheres fallen from the C@ACM.

10. EDS results of the membranes

The proportion of carbon element could reveal the change of carbon layer on the ceramic membrane. The EDS mapping results and the element percentage were shown in Figure S7. The proportion of C element was increased obviously after hydrothermal treatment at 180℃ due to the formation of the carbon layer. However, after recycled by calcination treatment at 550℃, the C atom proportion of the recycled ACM (0.26%) was almost the same as that of the raw ACM (0.71%). It indicated that the carbon layer was almost removed.

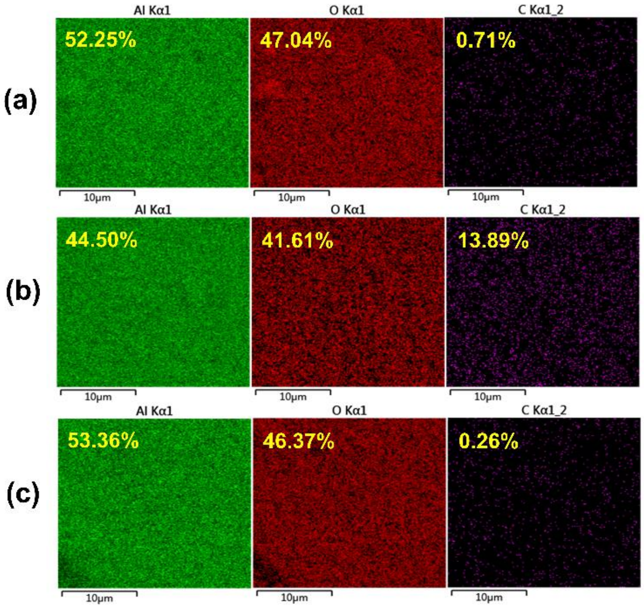

Figure S7. The EDS results of the membranes: (a) the raw ACM, (b) the C@ACM, (c) the recycled ACM.
